# Acute unilateral maculopathy associated with adult onset of hand, foot and mouth disease: a case report

**DOI:** 10.1186/s12886-019-1111-4

**Published:** 2019-05-07

**Authors:** Michael Reich, Bertan Cakir, Stevan Cvetkoski, Stefan J. Lang, Andreas Stahl, Thomas Ness, Hansjürgen Agostini, Clemens Lange

**Affiliations:** 1 Eye Center, Faculty of Medicine, Albert-Ludwigs University Freiburg, Killianstrasse 5, 79106 Freiburg, Germany; 2 Clinic for General and Visceral Surgery, Loerrach, Germany; 3 Department of Ophthalmology, University Medical Center Greifswald, Greifswald, Germany

**Keywords:** Acute maculopathy, Coxsackie virus, Hand, foot and mouth disease (HFMD), OCT-angiography, Posterior uveitis

## Abstract

**Background:**

To report the case of a 31-year-old patient with Hand, Foot and Mouth Disease (HFMD) and concurrent acute monocular maculopathy, and to describe multimodal imaging findings never before described including optical coherence tomography angiography (OCT-A).

**Case presentation:**

Nine days after the onset of clinically highly probable but not laboratory-verified HFMD, a 31-year old male noticed a central scotoma, distorted lines and loss of visual acuity (Snellen visual acuity 20/400) in his right eye. Funduscopy revealed focal alterations in the retinal pigmented epithelium (RPE) and yellow retinal dots corresponding to focal dots of decreased fundus autofluorescence (FAF) surrounded by increased FAF. Spectral domain optical coherence tomography (SD-OCT) demonstrated irregularities in the ellipsoide zone, hyperreflective dots above the RPE and RPE thickening. Fundus fluorescein angiography (FAG) revealed central hypofluorescence in the macular area in the early phase, as well as increasing focal hyperfluorescence in the late phase corresponding with RPE defects observed in FAF. Indocyanine green angiography (ICGA) showed central hypofluorescence in the early and late phase, corresponding with areas of reduced flow in the choroidea and choriocapillaris as apparent in OCT-A. Visual acuity improved within 3 months without any systemic or local therapy. At his three-month follow-up, SD-OCT revealed subtle subretinal fluid that resolved spontaneously over time. No signs of choroidal neovascularization were observed. Twelve months following the onset of symptoms Snellen visual acuity was 400/400. Multimodal imaging revealed subtly changed, decreased FAF while the choroidal architecture recovered completely as demonstrated by OCT-A.

**Conclusions:**

HFMD-associated maculopahty is an uncommon but important differential diagnosis of chorioretinitis with macular involvement. The prognosis can be good and the initially observed morphological pathologies such as impaired perfusion of the choroidal vessels can recover spontaneously over a period lasting 12 months. OCT-A can be employed as a non-invasive tool to detect the reduced perfusion of the choroidal vessels and for monitoring the disease course.

## Background

Hand, foot and mouth disease (HFMD) is a common and highly contagious infectious disease caused by Coxsackie viruses which belong to the Picornaviridae family of viruses. In Germany 80.000 to 140.000 cases are reported per year according to data of the Robert Koch Institut. It predominantly affects children and is characterized by fever and maculopapular or vesicular erupions on the hands, feet, and inside the mouth. Symptoms and rash normally disappear spontaneously within a week. However, systemic complications are possible such as gastrointestinal and respiratory symptoms, meningoencephalitis, and cardiac inflammation. Ocular complications such as conjunctivitis, keratoconjunctivitis, iridocyclitis, scattered hemorrhagic dots, and occlusive retinal vasculitis can rarely occur and result in temporary vision loss [1–9].

Here we report the case of a 31-year-old patient with HFMD and concurrent acute monocular maculopathy and describe multimodal imaging findings, including previously undescribed optical coherence tomography angiography (OCT-A).

## Case presentation

**Fig. 1:**
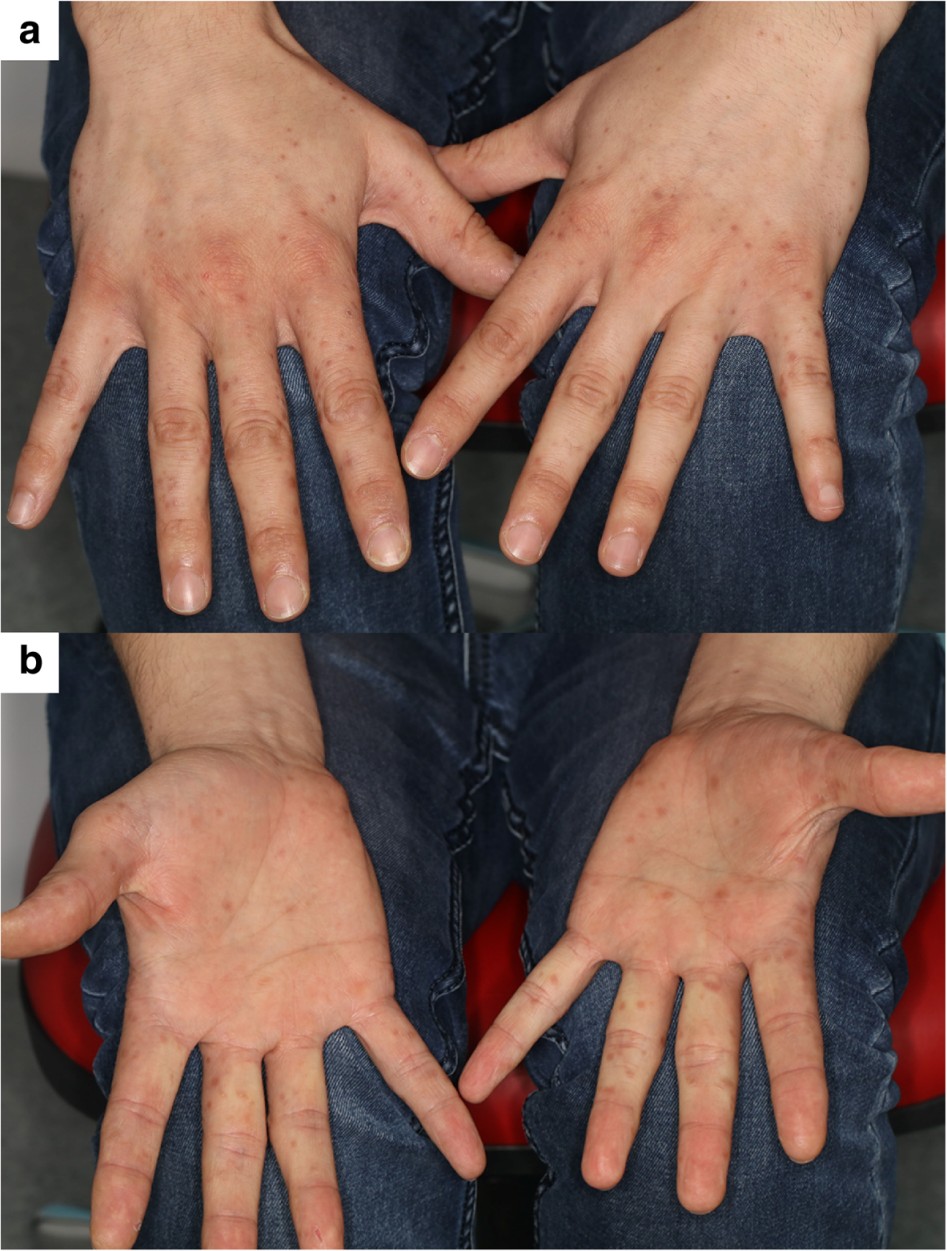
Erythema at baseline. Erythema with maculopapular and vesicular eruptions on the back (**a**) and the palm (**b**) of the hands which occurred 2 days before the beginning of ophthalmic symptoms

A 31-year-old, otherwise healthy man presented to our clinic with a 7-day history of central scotoma and metamorphopsia in his right eye. A local dermatologist had diagnosed an erythema involving maculopapular and vesicular eruptions on both hands, feet, and inside his mouth as HFMD 2 days before his visual symptoms began (Fig. 1a and b). A few days before the erythema’s onset, the patient had complained about severe fatigue, sore throat, fever, and chills. Two weeks before symptoms’ onset, his 4-year-old daughter presented similar symtoms and signs of HFMD.

**Fig. 2:**
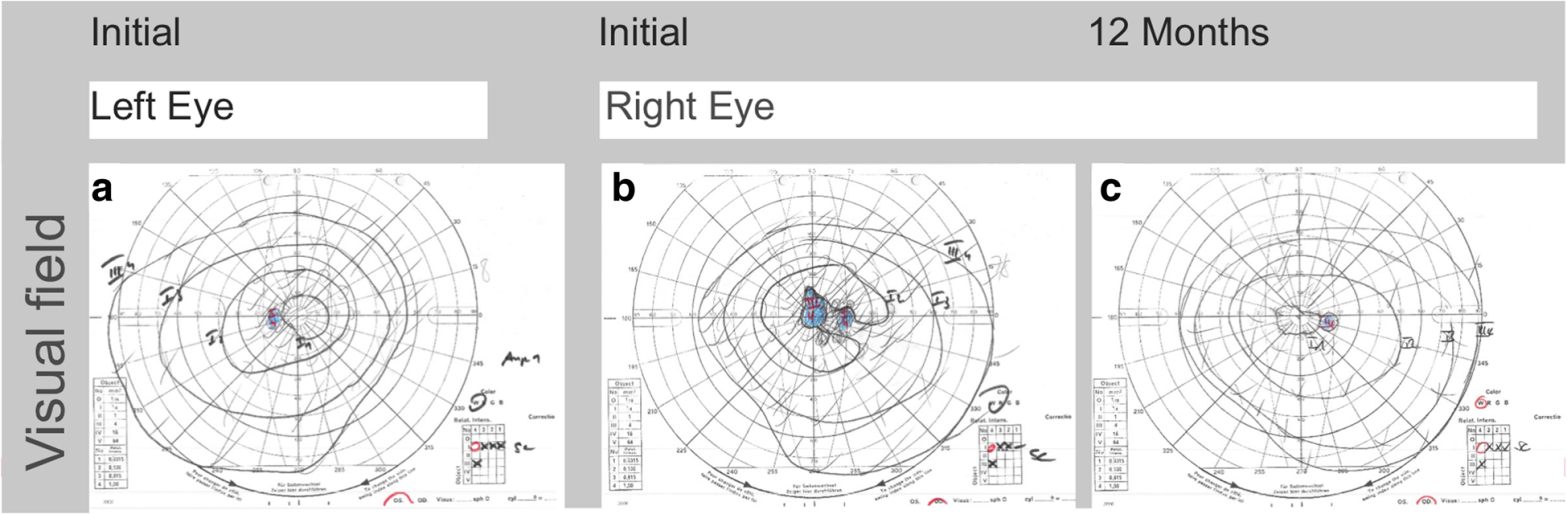
Visual field tests over a follow-up. Goldmann peripheral visual field testing showed, compared to the unaffected left eye (**a**), a central scotoma in the affected right eye at initial examination (**b**) which could not be detected anymore at the follow-up visit a year later (**c**)

**Fig. 3:**
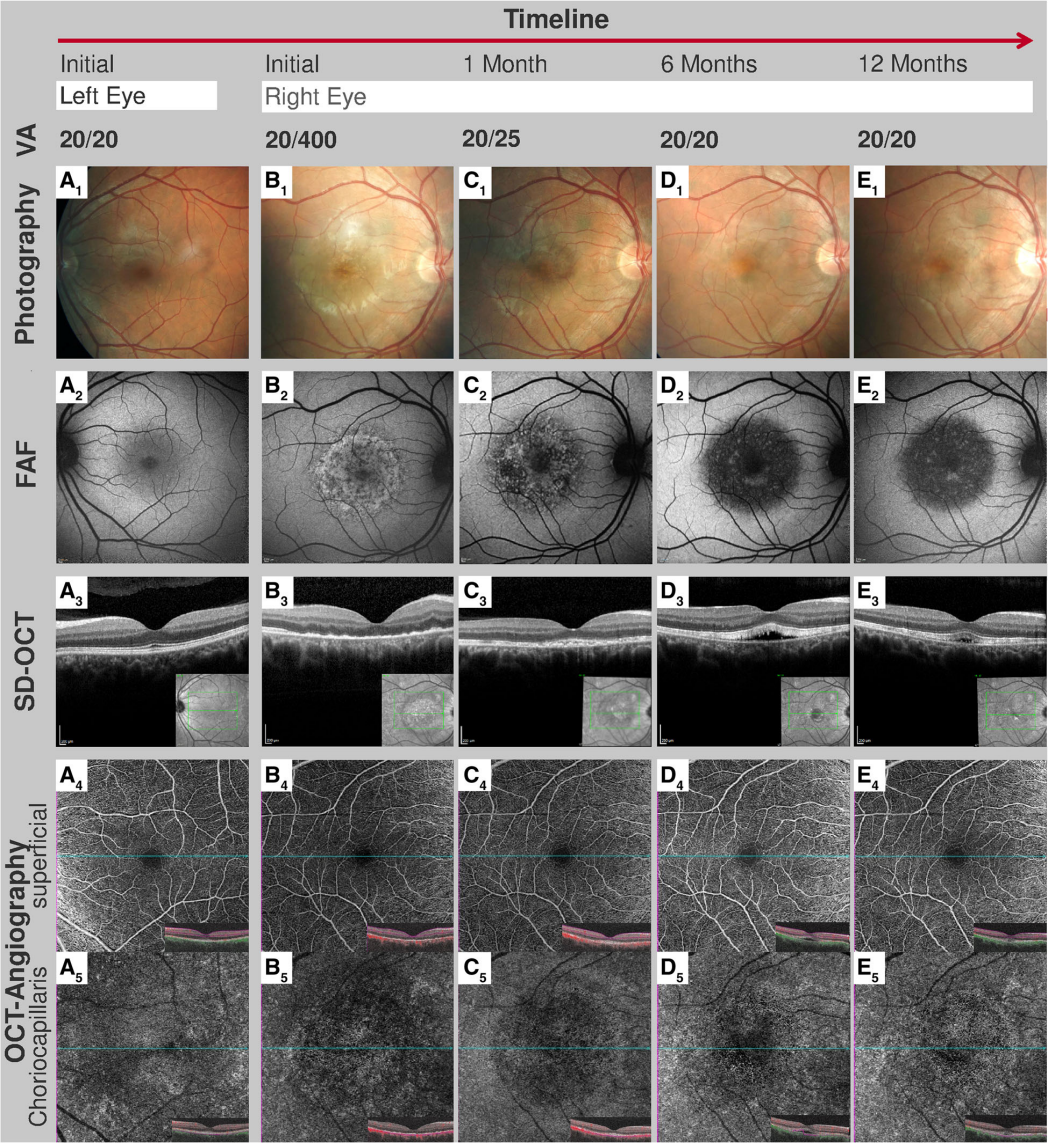
Clinical ophthalmological status over follow-up lasting 1 year. The findings of the unaffected left eye at initial examination are illustrated in A_1-5_. Visual acuity (VA) of the affected right eye normalized completely during the first 3 months. Color fundus photography demonstrated a central circular area of focal alterations of the retinal pigmented epithelium (RPE) and yellowish retinal dots (B_1_) which disappeared during follow-up (D_1_). Fundus autofluorescence (FAF) at baseline revealed dots of decreased FAF surrounded by increased FAF (B_2_) which resulted in completely decreased FAF over time (C_2_ + D_2_ + E_2_). Spectral domain optical coherence tomography (SD-OCT) showed irregularities in the ellipsoide zone, hyperreflective dots above the RPE, and RPE thickening (B_3_). RPE anomalies improved over the time (C_3_ + D_3_ + E_3_) but the subretinal fluid appeared 3 months after baseline, and first increased (D_3_) bevor decreasing (E_3_) without any therapy. Optical coherence tomography angiography (OCT-A) revealed a reduced flow in the choriocapillaris at initial examination (B_5_) - flow that improved over the next 3 months (C_5_ + D_5_) and nearly normal 1 year after initial examination (E_5_)

At his initial ophthalmologic examination, best corrected Snellen visual acuity was 20/400 in the right and 20/20 in the left eye. Intraocular pressure was 15 mmHg in both eyes. Goldmann peripheral visual field testing was within normal limits in the left eye (Fig. 2a) and revealed a central scotoma in the right eye (Fig. 2b). Slit lamp examination showed no cellular infiltration in the anterior chamber in either eye. Fundus examination of the right eye revealed central and parafoveal retinal pigment epithelium (RPE) irregularities and yellow retinal dots (Fig. 3B_1_). Fundus autofluorescence (FAF) demonstrated focal dots of decreased FAF surrounded by increased FAF corresponding to funduscopically detected focal alterations in the retinal RPE, and yellow retinal dots (Fig. 3B_2_). The left eye revealed no abnormalities. Spectral domain optical coherence tomography (SD-OCT) demonstrated irregularities in the ellipsoide zone, as well as hyperreflective dots in the RPE (Fig. 3B_3_). Fundus fluorescein angiography (FAG) exhibited central hypofluorescence in the macular area in the early phase, and focal hyperfluorescence in the late phase corresponding to RPE defects observed in FAF (Fig. 3B_1_). No macular exudation, extramacuar abnormalities or signs of vasculitis were observed. Indocyanine green angiography (ICGA) showed central hypofluorescence in the early and late phase (Fig. 3B_2_).

One month after initial ophthalmologic examination, visual acuity of the right eye improved spontaneously and reached 20/25 (left eye 20/20). Dermatological lesions subsided and only fingernail onychomadesis persisted. SD-OCT revealed persistent RPE changes (Fig. 3C_3_). The focal areas of increased autofluorescence changed to areas of decreased autofluorescence (Fig. 3C_2_) - changes that were associated with a subjectively improved vision and alleviated scotoma.

**Fig. 4:**
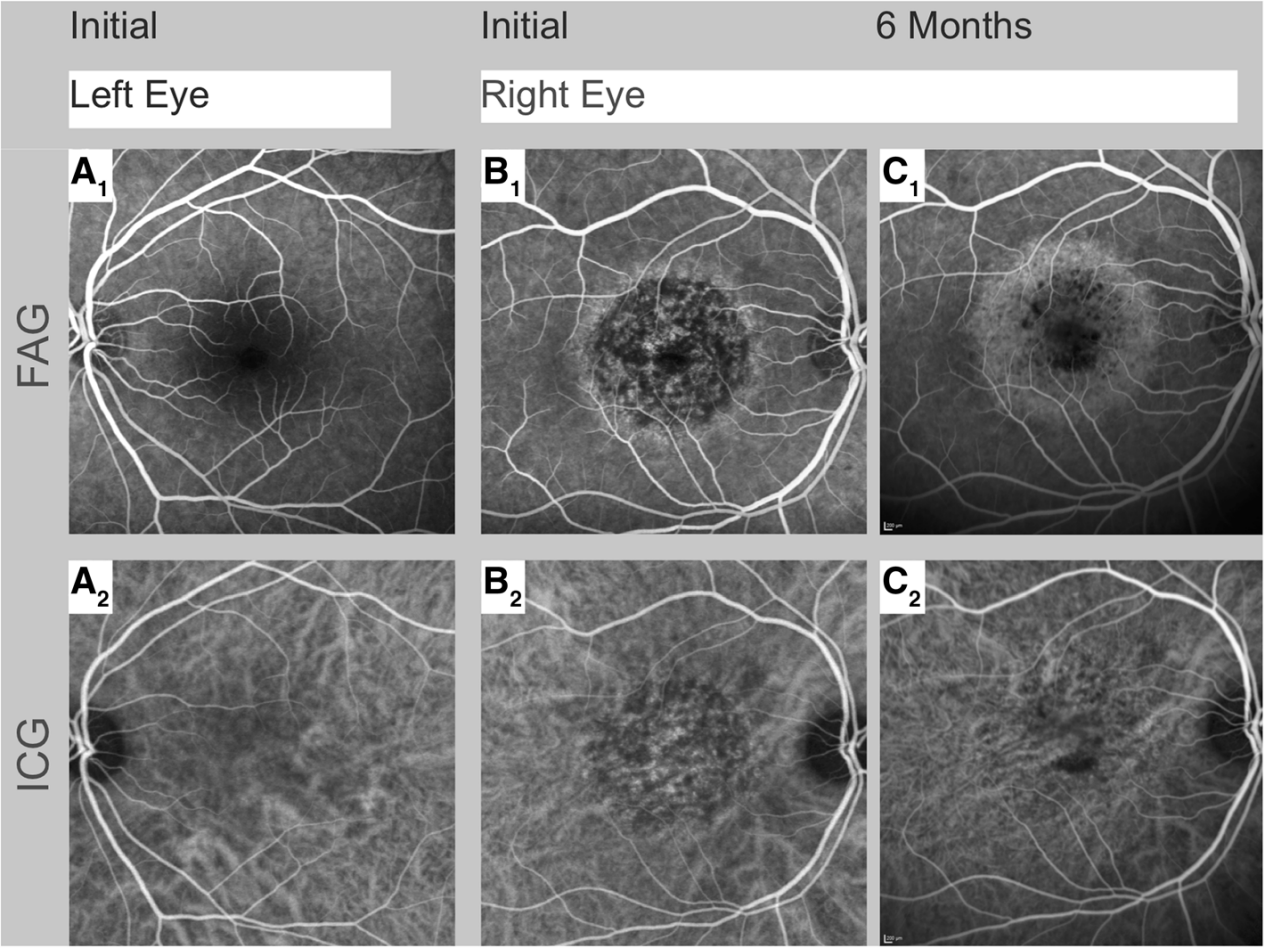
Fundus fluorescein angiography and indocyanine green angiography over a follow-up. The findings of the unaffected left eye at initial examination are illustrated in A_1+2_. Fundus fluorescein angiography (FAG) of the affected right eye showed central atrophy with no macular leakage, no extramacular abnormalities, and no vasculitis at initial examination and at follow-up after 6 months (B_1_ + C_1_). Indocyanine green angiography (ICGA) demonstrated central hypofluorescence in the early and late phases at initial examination and at follow-up after 6 months (B_2_ + C_2_)

Three months after his initial ophthalmologic examination, the patient was symptom-free. Interestingly, SD-OCT now revealed subretinal fluid, which increased until 6 months after the initial examination (Fig. 3D_3_). FAG und ICGA showed no signs of choroidal neovascularization (Fig. 4C_1 + 2_). Since the patient was symptom-free, no systemic or local therapy was prescribed. A year after his initial examination, the right eye’s visual acuity was stable at 20/20, there was no detectable scotoma (Fig. 2c), and the subretinal fluid had resolved spontaneously (Fig. 3E_3_) while FAF exhibited persistent areas of hypoautofluorescence (Fig. 3E_2_).

In addition to the conventional examinations, we conducted OCT-A (Zeiss Cirrus 5000 AngioPlex®) at his initial examination and at all follow-ups. Corresponding with the area of focal dots of decreased FAF surrounded by increased FAF and central hypofluorescence in the FAG and the ICGA, the choriocapillaris (CC), detected by OCT-A, revealed reduced flow at initial examination (Fig. 3B_5_). Over time, CC architecture recovered nearly completely (Fig. 3C_2_-E_2_), whereas anomalies in FAF, FAG and ICGA also changed, but remain.

## Discussion and conclusions

Viruses are frequent causes of infections involving the eye’s posterior segment. Measles, influenza, Ebstein-Barr and Rift Vallery fever virus can cause retinitis and chorioiditis, which is typically preceded by an acute systemic viral disease. Similarly, Coxsackie viruses infections can be associated with chorioretinitis as reported in just a few cases in the literature [1]. Diagnosing Coxsackie virus infection mainly relies on pathognomic clinical findings and can be supported by laboratory tests such as PCR [10]. Although our patient underwent no anterior-chamber or vitreous-fluid aspiration to detect the Coxsackie virus via PCR or cell culture, our patient’s clinical and funduscopic features are in line with previous case reports [1, 5, 9] and make the diagnosis of an HMFD-associated maculopathy very likely.

The underlying pathological mechanisms of HFMD-associated maculopathy remain enigmatic and may be explained by a direct viral infection of the outer retina and choroid, or by an autoimmune response.

Interestingly, the retinal changes observed in our case such as the irregularities in the photoreceptor layer and choroid were self-limiting. Visual acuity recovered in line with previous reports [1, 3, 4, 7]. Nevertheless, our patient demonstrated persistent focal areas of decreased FAF 12 months following disease onset, suggesting a permanent loss of RPE cells. This finding underlines the importance of FAF and OCT-A to monitor disease progression, and demonstrates the need for long-term follow-up to assess its progression and impact on visual acuity.

To the best of our knowledge, there are to date no reports describing the use of OCT-A to follow-up the choroidal changes in patients with HFMD-associated maculopathy. Our case demonstrates that choroidal flow on OCT-A was reduced at presentation and recovered almost completely over time. Reduced flow in OCT-A can be caused by decreased blood flow or by shadowing artifacts such as RPE clumping, the yellow retinal dots, or the accumulation of subretinal fluid [11]. However, since the reduced OCT-A flow in the CC was also present during follow-up examinations in areas without subretinal fluid and without RPE clumping, a shadowing artifact is unlikely. Similarly, our observation that the RPE changes persisted while the OCT-A signal improved significantly over time, also makes this artifact unlikely. Taken together, our observations favor the hypothesis of reduced blood flow in the CC as a primary or secondary cause of HFMD maculopathy, a hypothesis in line with findings demonstrating that HFMD can be associated with peripheral vasoconstriction [12] which may be caused by imbalanced vasoactive substances and cytokines following viral infection [13]. Our patient’s improved choroidal blood circulation during follow-up may also explain his good visual recovery despite persisting RPE changes.

To conclude: The Cocksackie virus is an uncommon but important differential diagnosis of chorioretinitis with atypical retinopathy. In this case, autofluorescence and OCT-A were useful non-invasive monitoring tools. Despite persisting morphological RPE-changes, our patient’s clinical and visual outcome was positive without systemic or local therapy.

## Abbreviations

CC: Choriocapillaris
FAF: Fundus autofluorescence
FAG: Fundus fluorescein angiography
HFMD: Hand, foot and mouth disease
ICGA: Indocyanine green angiography
OCT-A: Optical coherence tomography angiography
RPE: Retinal pigment epithelium
SD-OCT: Spectral domain optical coherence tomography
